# Comparison of Transient and Persistent Adverse Events After COVID-19 Vaccination: A Retrospective Analysis

**DOI:** 10.7759/cureus.63410

**Published:** 2024-06-28

**Authors:** Haruka Hikichi, Yuki Fujioka, Akiko Saga, Ken Watanabe, Ryo Hasegawa, Yuki Moritoki, Shigeharu Ueki

**Affiliations:** 1 Department of General Internal Medicine and Clinical Laboratory Medicine, Akita University Graduate School of Medicine, Akita, JPN; 2 Central Laboratory Division, Akita University Hospital, Akita, JPN

**Keywords:** post covid-19 vaccination, transient symptoms, persistent symptoms, adverse events following immunization, covid-19 vaccines, covid-19

## Abstract

Objective: Most reported adverse events following COVID-19 vaccination have been transient. However, persistent adverse events may occur with some frequency. This study aimed to analyze patient background characteristics and trends, with a focus on whether adverse events following COVID-19 vaccination were transient or persistent.

Methods: A retrospective study was performed at a single institution in Japan.

Patients: The study cohort included 47 patients who presented with symptoms after COVID-19 vaccination between May 2021 and September 2023. The patients were classified into two groups based on the duration of symptoms: transient group, less than four weeks; persistent group, greater than or equal to four weeks. Data on age, sex, body mass index, smoking history, underlying conditions, type of COVID-19 vaccination, number of doses, onset, symptoms, and treatments were collected retrospectively.

Results: The median age was 51.0 years and 74.5% were females, with a particularly high proportion of women in their 40s. The use of the bivalent omicron-containing booster vaccine (BA.1) was significantly more common in the persistent group than in the transient group (p = 0.0267). Onset in the transient group was more common after the first vaccination, whereas onset in the persistent group was more common after the second and subsequent vaccinations (p = 0.003). Regarding symptoms, pain was more frequent in the persistent group than in the transient group (60% vs. 13.6%; p = 0.001).

Conclusions: This study investigated the presence of persistent symptoms, especially pain, after COVID-19 vaccination. Persistent symptoms were frequently reported after the second vaccination. It should be noted that the study does not negate the usefulness of COVID-19 vaccines.

## Introduction

In 2020, the COVID-19 pandemic spread rapidly around the world, prompting urgent efforts to develop effective vaccines. In November of that year, Pfizer/BioNTech developed the BNT162b2 mRNA vaccine [1]. Subsequently, the mRNA-1273 vaccine (Moderna) [2] and the Nuvaxovid COVID-19 vaccine (Takeda), a protein-based adjuvanted vaccine, were approved for emergency use in Japan, starting in February 2021. As the virus continued to mutate, booster vaccines targeting specific variants, such as the bivalent omicron-containing booster vaccine (BA.1), were introduced, followed by vaccines targeting BA.4-5 and later the XBB.1.5 lineage [3,4].

Studies conducted in the United States and Japan have consistently demonstrated the safety of COVID-19 vaccines, with no increased risk of mortality associated with vaccination [5,6]. However, studies on non-fatal adverse events have produced mixed results. While short-term studies have predominantly reported minor reactions such as local pain and fatigue within seven days of vaccination [1,2,7-12], a post-trial analysis revealed a 16% higher risk of serious adverse events for mRNA vaccines compared with placebo [13]. Furthermore, post-marketing surveillance has identified cases of autoimmune disorders and serious adverse events following vaccination, raising concerns about the long-term safety profile of COVID-19 vaccines [14-22]. Despite the growing body of literature on adverse events following vaccination, comparisons between transient and persistent symptoms remain unclear.

In Japan, patients who experience an adverse event after COVID-19 vaccination initially visit a familiar clinic or the medical institution where they received the vaccination. If they have refractory symptoms, they are referred to a medical institution in the prefecture that serves as a consultation center.

This retrospective observational study was conducted in a northern Japanese prefecture with a population of 900,000. The COVID-19 vaccination coverage in the prefecture was the highest in the country, with first vaccination coverage of 87.7%, second vaccination coverage of 86.9%, third vaccination coverage of 78.8%, and fourth vaccination coverage of 62.7%, as published by the government at the end of February 2024.

Patients who were referred to the only consultation center in the prefecture were enrolled in the study. The study aimed to determine whether post-vaccination symptoms were transient or persistent and to clarify the background characteristics and trends in the groups of patients with transient or persistent symptoms.

## Materials and methods

In the present study, we conducted a retrospective analysis at the only COVID-19 vaccine adverse event consultation center in our prefecture. We identified a total of 51 cases with age ≥15 years between May 2021 and September 2023. After carefully reviewing the medical records, we found four cases in which symptoms of pre-existing conditions worsened or new diagnoses were made after vaccination, comprising cases of polymyalgia rheumatica (n = 2), malignant lymphoma (n = 1), and brain tumor (n = 1). These cases were considered inappropriate for inclusion as post-vaccination symptoms and were excluded from the study. The remaining 47 cases were followed for 12 weeks to assess post-vaccination symptoms. Patients whose symptoms improved within four weeks were classified as the transient group (n = 22), while patients whose symptoms persisted for greater than or equal to four weeks were classified as the persistent group (n = 25). Data on age, sex, body mass index, smoking history, underlying conditions, type of COVID-19 vaccination, number of doses, onset, symptoms, and treatments were collected. Statistical analyses were performed using Welch's t-test or the chi-square test. The study was approved by the Institutional Review Board of Akita University Graduate School of Medicine (No.: 3080).

## Results

**Figure 1:**
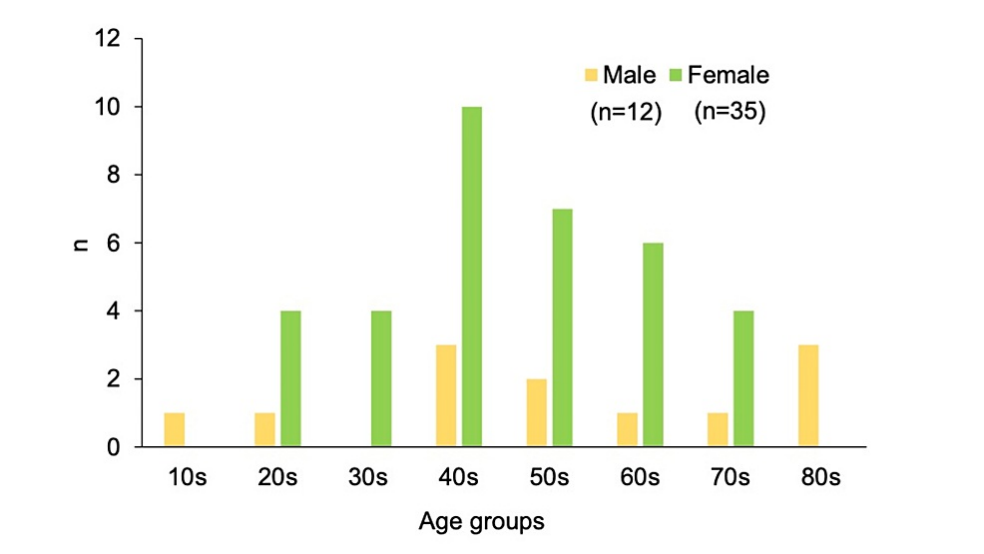
Sex differences across age groups in the study cohort. The bar graph illustrates the distribution of the 47 patients by age group, ranging from teenagers to octogenarians.

The median age of all 47 patients was 51.0 years. The transient group had a median age of 46.5 years (20-85 years) and the persistent group had a median age of 55.0 years (15-89 years), with no significant difference between the two groups by Welch’s t-test (Table 1). Of the total cases, 74% were females, comprising 86% in the transient group and 64% in the persistent group. The transient group contained a higher proportion of women, but there was no significant difference by the chi-square test (Table 1). By age group, the largest proportion were people in their 40s, with a particularly high proportion for women (Figure 1).

**Table 1:** Characteristics of patients with adverse events after COVID-19 vaccination.

Characteristics	Transient group (N = 22)	Persistent group (N = 25)	p-value
Age, median (range)	46.5 (20-85)	55.0 (15-89)	0.35
Female, N (%)	19 (86)	16 (64)	0.08
BMI, mean (range)	22.4 (15.9-29.1)	24.2 (16.5-33.2)	0.18
Smoking habit, N (%)	2 (9.0)	7 (28.0)	0.10
Any comorbidities, N (%)	18 (82)	24 (96)	0.12
Type of vaccination			
BNT162b2, N (%)	10 (45)	7 (28)	0.11
Omicron-containing booster vaccine (BA.1), N (%)	0 (0)	5 (20)	0.027
mRNA-1273, N (%)	5 (23)	6 (24)	1.00
Unknown, N (%)	7 (32)	7 (28)	
Vaccination dose at the onset			
1^st^, N (%)	10 (45)	2 (8)	0.003
2^nd^, N (%)	8 (36)	10 (40)	0.080
3^rd^ or beyond, N (%)	4 (18)	13 (52)	0.016

**Figure 2:**
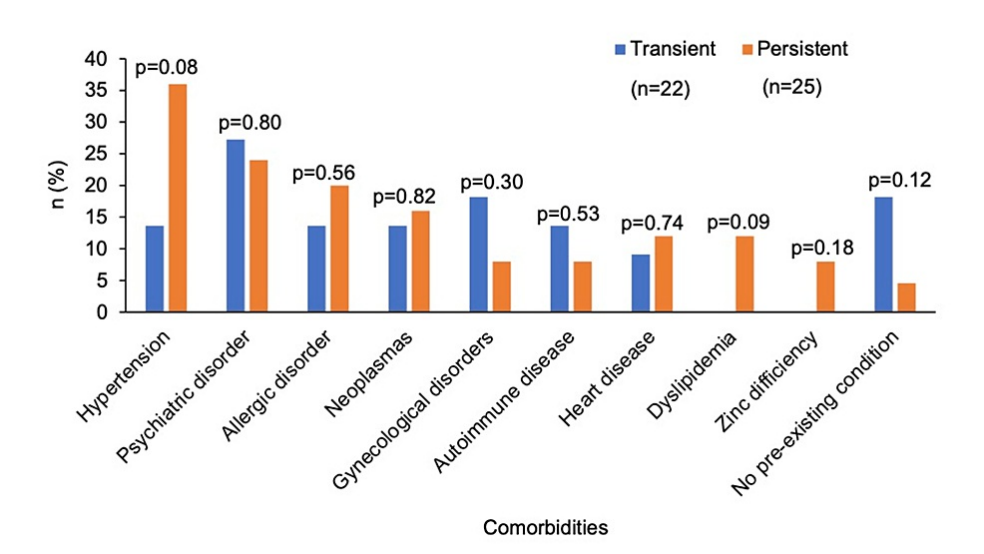
Comorbidities in the transient and persistent groups. The graph illustrates the percentages of underlying medical conditions in the groups.

In the total cohort, 89% had some type of underlying condition, most commonly hypertension (25.5%), psychiatric disorders (25.5%), and allergic diseases (17%). There were no significant differences for each of these diseases between the persistent and transient groups (Figure 2).

The type of COVID-19 vaccine was the BNT162b2 vaccine in 36%, bivalent omicron-containing booster vaccine (BA.1) in 11%, mRNA-1273 vaccine in 23%, and unknown in 30%. Compared with the transient group, the frequency of the bivalent omicron-containing booster vaccine (BA.1) was significantly higher in the persistent group (p = 0.027) (Table 1).

**Figure 3:**
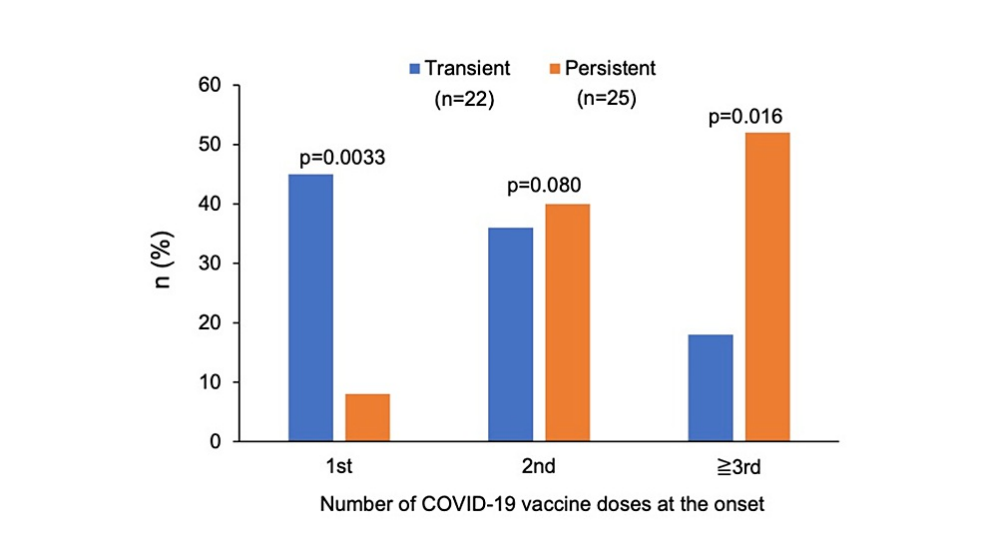
The number of COVID-19 vaccine doses at the onset of adverse events in the transient and persistent groups, categorized into first dose, second dose, and third or subsequent doses.

For adverse events related to the number of vaccinations, the percentage after the first vaccination in the transient group was 45%, which was significantly higher than that in the persistent group (p = 0.003). The incidence in the transient group tended to decrease as the number of vaccinations increased. However, the incidence in the persistent group tended to increase as the number of vaccinations increased: 8% after the first vaccination, 40% after the second vaccination, and 52% after the third or subsequent vaccinations. For the third and subsequent vaccinations, the incidence in the persistent group was higher than that in the transient group, with a significant difference (p = 0.016) (Figure 3).

**Figure 4:**
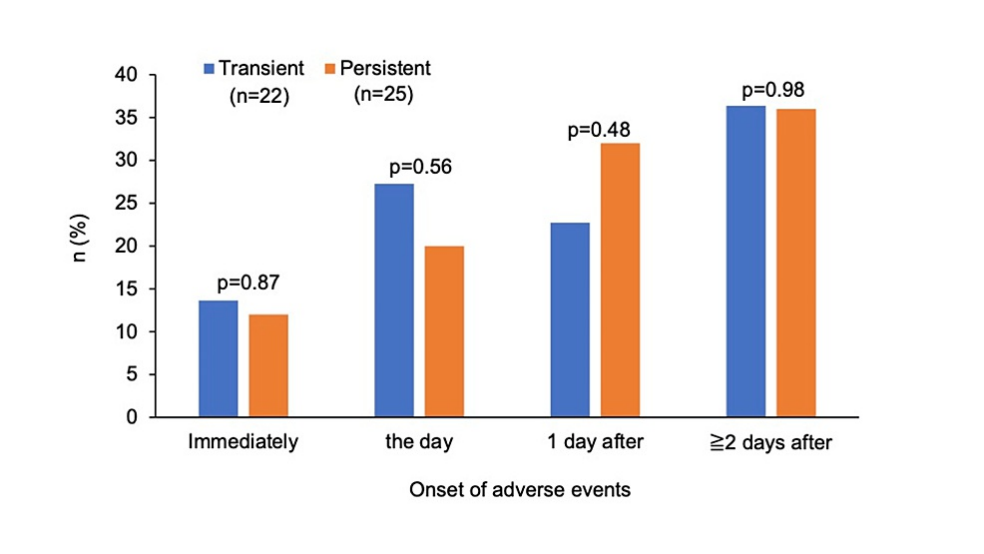
The time of onset of adverse events in the transient and persistent groups. The graph illustrates the proportions of the time of onset of adverse events after vaccination in the groups, categorized into immediately, the day of vaccination, one day after vaccination, and ≥ two days after vaccination.

Symptom onset in the persistent group occurred immediately after vaccination in 12%, on the day of vaccination in 20%, after one day in 32%, and after ≥ two days (maximum, one month) in 36%, with an increasing trend over time (Figure 4). In the transient group, the onset occurred immediately after vaccination in 14%, on the day of vaccination in 27%, after one day in 23%, and after ≥ two days (maximum, 11 days) in 36%, with no trend over time. There were no significant differences between the transient group and the persistent group when onset was immediately (14% vs. 12%) (p = 0.87), the day of vaccination (27% vs. 20%) (p = 0.56), one day after vaccination (23% vs. 32%) (p = 0.48), or ≥ two days after vaccination (36% vs. 36%) (p = 0.98).

**Figure 5:**
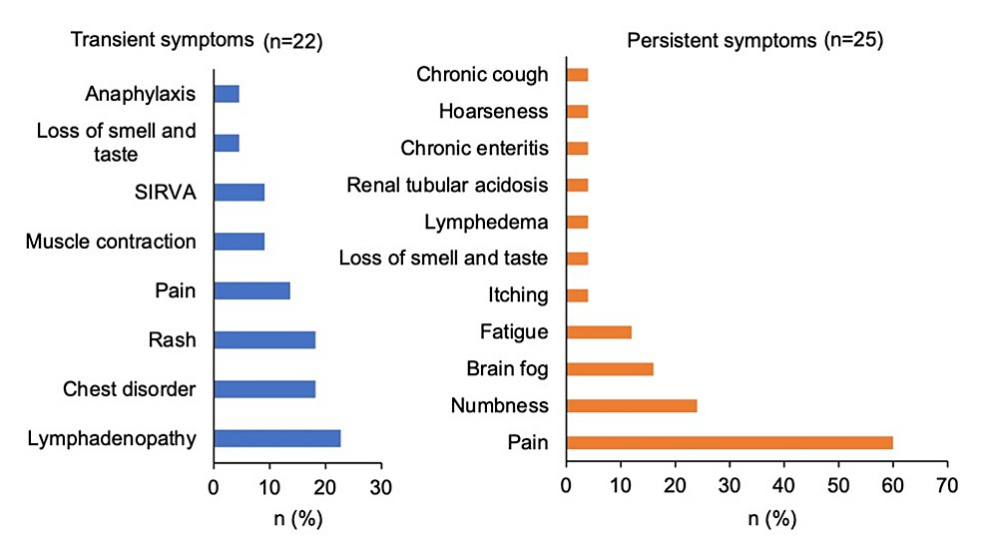
Proportions of adverse event symptoms in the transient and persistent groups. SIRVA: shoulder injury related to vaccine administration.

**Figure 6:**
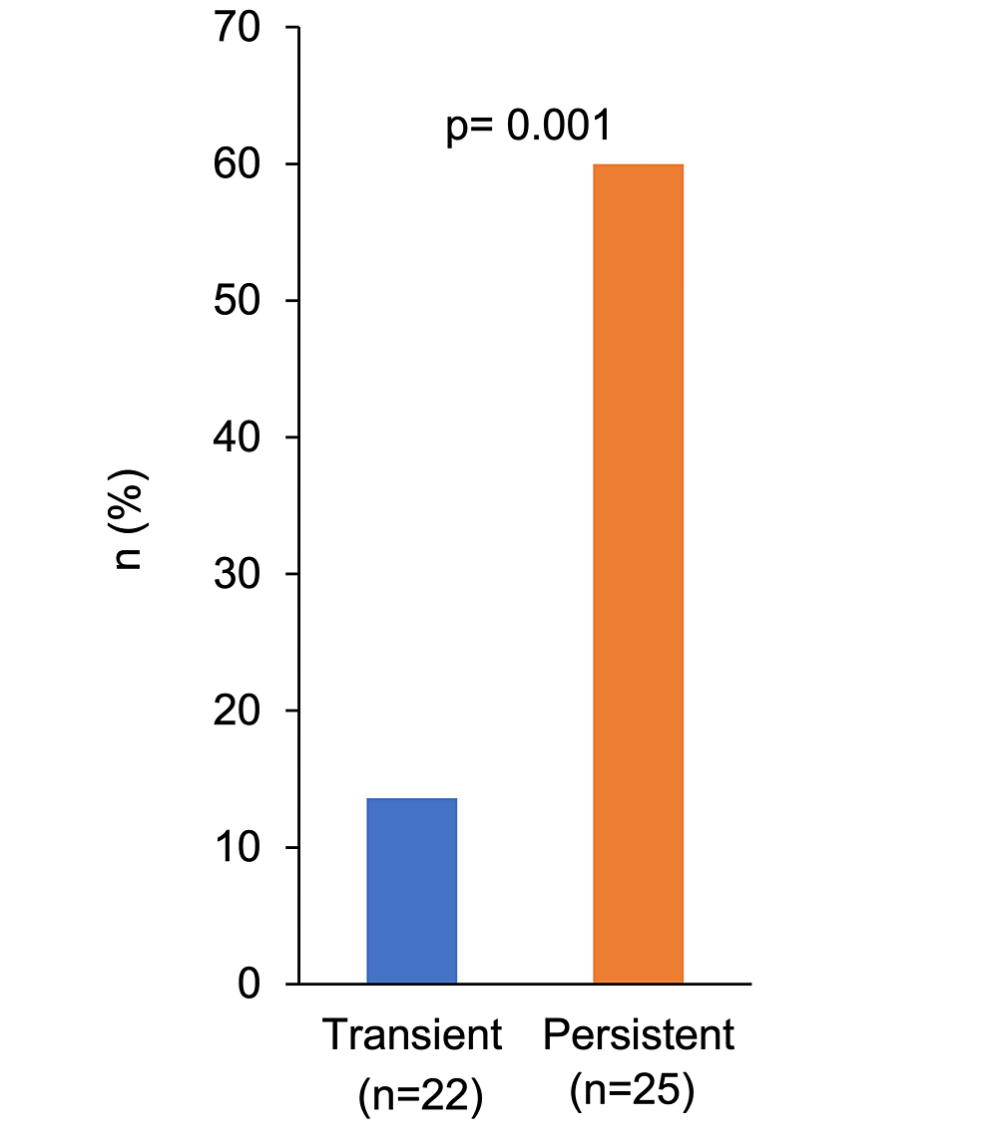
Proportions of pain in the transient and persistent groups.

A variety of symptoms were observed in each of the two groups (Figure 5). The only two symptoms found in both the transient and persistent groups were loss of smell and taste and pain. A comparison of the frequency of these two symptoms showed no significant difference for loss of smell and taste (p = 0.93). However, pain was more frequent in the persistent group (60% vs. 13.6%) (p = 0.001) (Figure 6). In the persistent group, 67% (n = 10) observed pain at the non-inoculated site and 33% (n = 5) at the inoculated site. In the transient group, 100% (n = 3) observed pain at the non-inoculated site. In patients who complained of pain, the medical record confirmed that there was no pain in the same area before the vaccination.

Regarding medications, 27% (n = 6) of the transient group and 100% (n = 25) of the persistent group required medication, with a significant difference (p = 0.003). Medications were chosen according to individual symptoms, with the transient group using antiallergics (13.6%), herbal medicines (9.1%), and topical steroids (4.5%), and the persistent group using analgesics (48%), herbal medicines (24%), zinc preparations (12%), systemic steroids (4%), antidepressants (4%), iron preparations (4%), and folic acid preparations (4%).

## Discussion

Regarding the age and sex aspects of adverse events following COVID-19 vaccination, previous studies in Nigeria and Nepal reported no association between age and adverse events, while a study in Korea found that adverse events became less frequent with older age [23-25]. Meanwhile, a one-year observational study in northern India found that individuals aged ≥40 years were more likely to experience adverse events, with women being 1.78 times more susceptible [26]. Moreover, a study on subacute thyroiditis associated with COVID-19 vaccination found that 74.5% of affected individuals were females and the median age was 39.5 years [22]. In the present study, the proportion of women in their 40s tended to be significantly higher. This finding supports the notion that women in their 40s may be at risk of developing adverse events following COVID-19 vaccination.

For persistent adverse events, the study from India further found 1.66 times higher odds for persistent adverse events (median duration: 120 days) in women, although there were no significant differences by age [26]. In the present study, the persistent group tended to have a lower proportion of women and a slightly higher median age compared with the transient group, but no significant differences were found between the two groups. The results may have differed because the previous study in India was a large prospective observational study, in which the population was the total COVID-19 vaccinated population, whereas the present study included only patients who developed adverse events.

Reports on clinical trials of COVID-19 vaccines have indicated no significant differences between the incidences of adverse events after the first and second vaccinations, other than a trend toward a higher incidence of fever after the second vaccination [1,2,27]. Among post-approval reports, we did not find any studies reporting differences in adverse events according to the number of vaccinations. In the present study, the transient group had a higher incidence of adverse events after the first vaccination, whereas the persistent group showed a trend toward a higher incidence of adverse events after the second or third vaccination. There may be a relationship between additional vaccinations and the persistence of symptoms after vaccination.

A study in Jordan reported that 35% developed adverse events nine to 12 hours after vaccination, while a study in Nigeria reported that 62.2% developed adverse events within 24 hours of vaccination [23,28]. These studies suggest that adverse events following COVID-19 vaccination predominantly occur within a relatively short period of time. In the present study, 36% developed adverse events on the day of vaccination, 28% one day after vaccination, and 36% at ≥ two days after vaccination, suggesting that a proportion of cases developed adverse events at ≥ two days. The previous studies included first and second doses, whereas the present study included a wide range from first to multiple doses, and this may have led to the differences in the findings.

There are few reports of persistent non-specific symptoms after COVID-19 vaccination. A study in India found that approximately 4% of vaccine recipients complained of persistent musculoskeletal disorders (e.g., knee or back pain), and had a median duration of hospitalization of four months [26]. Meanwhile, a study in Jordan found that 41.2% had vaccine site pain and 17.5% had general body pain [28]. In the present study, 60% of patients with persistent symptoms reported pain, suggesting a possible association between pain and COVID-19 vaccination.

Treatments for adverse events have not been established. In a study in Nigeria, 46.7% of people with adverse events experienced mild relief without treatment, and 36.1% used paracetamol. Individuals whose adverse events resolved within three days tended not to use drugs, while those whose adverse events did not resolve within three days tended to use drugs, suggesting a significant association between symptom duration and drug use [23]. The results of the present study were similar, with 100% of subjects in the persistent group requiring medication, particularly analgesics, compared with only 27% in the transient group.

The present study had some limitations. It was a small retrospective study of Japanese patients, the population was not identified, and the risk factors were not analyzed. Larger prospective studies are needed.

## Conclusions

The present study indicates that women in their 40s may be at risk of developing adverse events following COVID-19 vaccination. Transient symptoms were more common after the first vaccination, while persistent symptoms were more frequent after the second vaccination. The most common persistent symptom was pain requiring the use of long-term analgesics. This study has demonstrated that the adverse events following COVID-19 vaccination were not always transient and that some patients presented with persistent symptoms. Overall, COVID-19 vaccines are very safe, and widespread vaccination has saved many lives. It is important to remember this and continue to provide accurate information about vaccines.
